# Recurrent Oncocytoma of the Lacrimal Sac

**DOI:** 10.1155/2022/2955030

**Published:** 2022-02-28

**Authors:** Faris Almutairi, Mazen Alsamnan, Azza Maktabi, Sahar Elkhamary, Hind M. Alkatan, Humoud AlOtaibi

**Affiliations:** ^1^Department of Oculoplastic Surgery, King Khaled Eye Specialist Hospital, Al Urubah Road, Umm Al Hamam Al Gharbi, Riyadh 11462, Saudi Arabia; ^2^Department of Pathology, King Khaled Eye Specialist Hospital, Al Urubah Road, Umm Al Hamam Al Gharbi, Riyadh 11462, Saudi Arabia; ^3^Diagnostic Imaging Department, King Khaled Eye Specialist Hospital, Al Urubah Road, Umm Al Hamam Al Gharbi, Riyadh 11462, Saudi Arabia; ^4^Department of Ophthalmology and Pathology, King Saud University, College of Medicine, Riyadh, Saudi Arabia

## Abstract

Oncocytoma of the lacrimal sac is an extremely rare tumor. In this report, we present the case of an 82-year-old woman who presented with swelling in the region of the lacrimal sac. Systemic examination and ophthalmic examination of both eyes were unremarkable. Computed tomography of the brain and orbits revealed a mass lesion involving the right lacrimal sac with expansion of the related nasolacrimal duct. Neither bone destruction nor tissue invasion was observed. Right external dacryocystectomy and debulking of the tumor were performed. Histopathological examination of the surgical specimen showed oncocytic cells arranged in an adenomatous fashion, and a diagnosis of benign oncocytoma was made. Three years later, the same patient presented with a similar complaint that was pathologically proven to be a recurrent benign oncocytoma of the lacrimal sac.

## 1. Introduction

Oncocytic tumors originate from epithelial cells and form secondary to cell aging or functional exhaustion. Oncocytic cells exhibit numerous mitochondria with characteristic histological features of a swollen granular eosinophilic cytoplasm. These cells have a preserved ability to multiply and form daughter cells that maintain their oncocytic features [1], and oncocytoma, first defined by Jaffe in 1932, is a benign tumor that consists predominantly of oncocytes [2]. They rarely arise outside the salivary glands, kidneys, and the thyroid gland; however, oncocytoma/adenoma is the most frequently diagnosed form of oncocytic neoplasms. Recurrence after surgical excision is extremely rare; to our knowledge, only one case has been reported in the literature of oncocytoma that recurred after partial excision of the tumor.

In this report, we describe the case of an 82-year-old woman who underwent right external dacryocystectomy for oncocytoma of the lacrimal sac a few years earlier and presented again with a recurrent lesion. Thorough clinical, radiological, and pathological examinations were performed, and the diagnosis of recurrent benign oncocytoma was made.

## 2. Case Presentation

**Figure 1:**
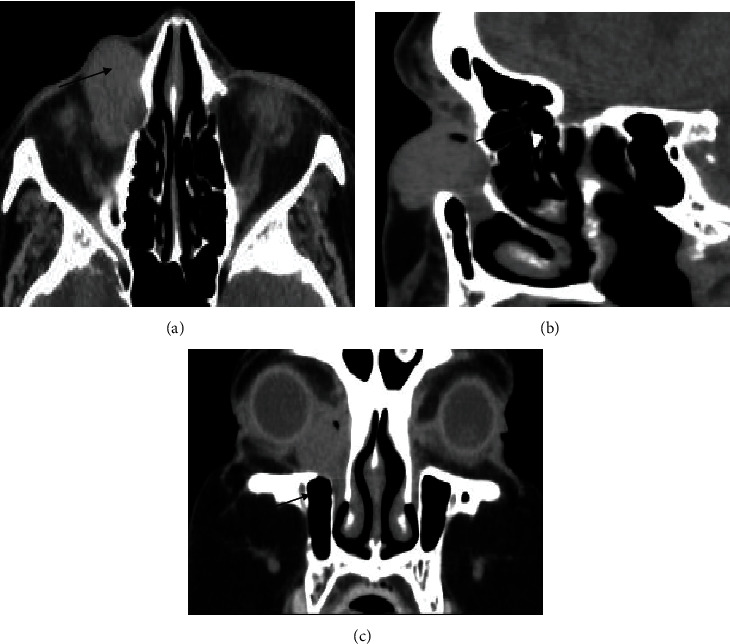
(a–c) Orbital computed tomography demonstrating an oval, solid, well-circumscribed, homogeneous mass extending from the lacrimal sac into dilated duct without any evidence of invasion into adjacent bones. (c) Coronal CT scan before the second excision.

An 82-year-old woman presented with swelling over the region of the right lacrimal sac for one year. Her medical history was significant only for diabetes mellitus type 2. Upon examination, the lesion was soft and bluish, with no tenderness or other signs of inflammation in the region. The fluorescein dye disappearance test was negative in both eyes. Irrigation showed patent canaliculi and nasolacrimal ducts. The patient underwent computed tomography (CT) of the brain and orbits, which showed evidence of a well-defined mass lesion on the right nasolacrimal sac with a density approaching 78 HU. The mass was approximately 2.7 × 2.0 cm and mildly extended and expanded into the related part of the right nasolacrimal sac with a widening of the related part of the right nasolacrimal duct without any evidence of bone destruction or tissue invasion (Figures 1(a) and 1(b)). It had a pocket of air density along its extreme nasal aspect, with a mild molding appearance of the related part of the globe. There was mild thickening at the retrobulbar end surrounding the fat. Magnetic resonance imaging (MRI) was recommended for further assessment and characterization; however, our patient was not fit for MRI because of previous knee replacement surgery. These findings were discussed with the patient, and surgical resection in the form of dacryocystectomy was planned.

**Figure 2:**
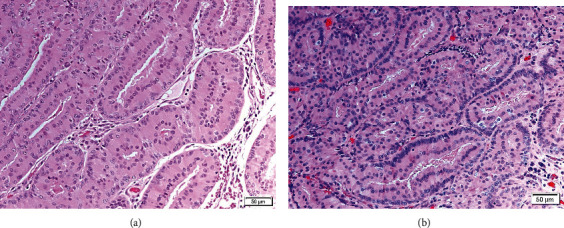
Sheaths of oncocytes, which are modified long epithelial cells characterized by abundant eosinophilic cytoplasm, arising in an adenomatous pattern. Hematoxylin and eosin. (a) Section of the primary tumor; (b) section of the recurrent tumor.

The patient underwent right external dacryocystectomy and debulking of the tumor as it extended beyond the right lacrimal sac; therefore, an attempt to resect all accessible parts of the tumor was performed and sent for pathologic assessment. Postoperative recovery was uneventful. Histopathological examination of the surgical specimen revealed epithelial cells arranged in an adenomatous fashion and in cords (Figure 2(a)). The epithelial cells lining the acini were relatively benign and moderately eosinophilic. No mitotic figures or atypia was observed. The patient was diagnosed with oncocytoma of the right lacrimal sac. The surgical specimen underwent further examination by immunohistochemistry which showed the expression of CK18 in inner cells and P63 in outer cells.

**Figure 3:**
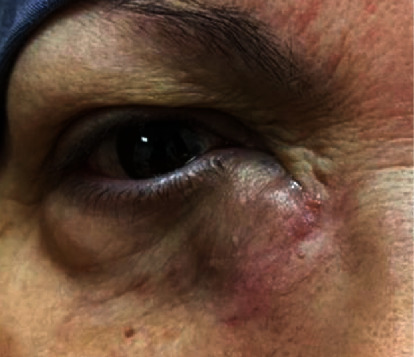
Photograph showing a swelling below the right medial canthal tendon with bluish discoloration of the overlying skin.

Three years later, our patient presented to the clinic with a complaint of swelling over the right lacrimal sac region that had been growing over the course of eight months (Figure 3). CT of the brain and orbits revealed a similar appearance to the previously mentioned mass lesion along the right lacrimal sac with an extension to the preseptal area (Figure 1(c)). Excisional biopsy was performed under local anesthesia, which was confirmed to be recurrent oncocytoma of the lacrimal sac after histopathological examination (Figure 2(b)). The patient was seen as a follow-up one month postoperatively; she was doing fine and has no complaints.

## 3. Discussion

Oncocytic lesions can be hyperplasia, metaplasia, or neoplasia. This article reports the case of a rare benign neoplastic lesion of the lacrimal sac. The clinical features of lacrimal sac oncocytoma range from being asymptomatic to epiphora and chronic dacryocystitis [3–4]. It has also been found incidentally during dacryocystorhinostomy for nasolacrimal duct obstruction [5]. In our case, the patient was treated in the oculoplastic clinic with a complaint of a mass noticed over the region of the right lacrimal sac, which gradually increased in size.

Oncocytoma of the ocular adnexa more commonly involves the caruncle. A literature review by Say et al. showed that 59% of 212 cases of oncocytic lesions were in the caruncle, 19% were in the lacrimal sac, and only 5% were in the lacrimal gland. The remaining lesions were in the eyelids and conjunctiva. They also have observed that oncocytoma mostly presents in patients older than 40 years and has a duration of symptoms of more than 1 year [3]. Another literature review by Calle et al. [5] summarized the data of eight patients diagnosed with oncocytic tumors in the lacrimal gland and found that five cases were of oncocytoma and three were malignant. The duration of symptoms ranges from 1 to 3 months for malignant oncocytic tumors and 2-96 months for oncocytoma. Calle et al. [5] also included the youngest patient recorded (18 months old) with oncocytoma [6]. Recurrence was reported in one patient with an oncocytoma that was partially excised surgically.

In general, MRI provides information on anatomical tumor extent, margins, angulation, and configuration of the lacrimal gland fossa mass, while CT is considered the imaging modality of choice to assess bony changes and calcification [7]. Benign oncocytic neoplasms of the orbit typically appear solid, well-circumscribed, oval, round-edged, hyperdense, well-defined, homogenous in texture, and with enhancement on CT and MRI [8]; however, in some cases, they may also contain mixed cystic and solid components. In contrast to benign lesions, malignant tumors demonstrate irregular margins with nodularity and infiltration into the adjacent orbital fat [9]. In the present case, the tumor did not show any changes in the adjacent bones.

The diagnosis is typically based on microscopic evidence of cellular changes that predominantly exhibit oncocytic features. Oncocytosis is known to occur secondary to the degenerative and aging process of epithelial cells. Several mitochondrial DNA mutations have been found to play a role in the transformation of epithelial cells into oncocytic cells. A nationwide study by Østergaard et al. provided the clinicopathological characteristics of oncocytic lesions, which supports the fact that oncocytic lesions have similar immunohistochemical findings to their original cells [10]. This can be achieved by assessing the expression of cytokeratin (CK), a protein of intermediate filaments found in epithelial cells; 20 subtypes have been identified, and they vary in expression between different tissues [11]. In the present case, immunohistochemistry showed the expression of CK18 in inner cells and P63 in outer cells. Mulay et al. reported a case of oncocytoma of the lacrimal sac which was reactive to pan-CK, CK7, and CK19 [12].

Complete surgical resection is the treatment of choice for oncocytoma with long-term follow-up for local recurrence. To our knowledge, recurrence after complete surgical resection has not yet been reported. In our case, complete surgical excision was not feasible because the tumor was large and extended beyond the lacrimal sac. The patient presented again three years later with a similar picture of a swelling below the medial canthal tendon of the right side that had been growing for eight months. Postcontrast CT scans showed a similar appearance to the previous mass extending to the preseptal area; therefore, excisional biopsy was performed.
